# Stromal Cell-Derived Factor-1α Plays a Crucial Role Based on Neuroprotective Role in Neonatal Brain Injury in Rats

**DOI:** 10.3390/ijms160818018

**Published:** 2015-08-05

**Authors:** Miki Mori, Keiichi Matsubara, Yuko Matsubara, Yuka Uchikura, Hisashi Hashimoto, Toru Fujioka, Takashi Matsumoto

**Affiliations:** Department of Obstetrics and Gynecology, Ehime University School of Medicine, Toon, Ehime 791-0295, Japan; E-Mails: morimiki@m.ehime-u.ac.jp (M.M.); takeyu@m.ehime-u.ac.jp (Y.M.); yuka.itani@gmail.com (Y.U.); hashimoto.hisashi.mm@ehime-u.ac.jp (H.H.); fujioka@m.ehime-u.ac.jp (T.F.); matsugen@m.ehime-u.ac.jp (T.M.)

**Keywords:** hypoxic-ischemic encephalopathy, magnetic resonance imaging, morris water maze test, rat, Rotarod test, stromal cell-derived factor-1α, TTC staining

## Abstract

Owing to progress in perinatal medicine, the survival of preterm newborns has markedly increased. However, the incidence of cerebral palsy has risen in association with increased preterm birth. Cerebral palsy is largely caused by cerebral hypoxic ischemia (HI), for which there are no effective medical treatments. We evaluated the effects of stromal cell-derived factor-1α (SDF-1α) on neonatal brain damage in rats. Left common carotid (LCC) arteries of seven-day-old Wistar rat pups were ligated, and animals were exposed to hypoxic gas to cause cerebral HI. Behavioral tests revealed that the memory and spatial perception abilities were disturbed in HI animals, and that SDF-1α treatment improved these cognitive functions. Motor coordination was also impaired after HI but was unimproved by SDF-1α treatment. SDF-1α reduced intracranial inflammation and induced cerebral remyelination, as indicated by the immunohistochemistry results. These data suggest that SDF-1α specifically influences spatial perception abilities in neonatal HI encephalopathy.

## 1. Introduction

Hypoxic-ischemic encephalopathy (HIE), a complex condition that involves several inflammatory events, is a major cause of neonatal death and long-term disability. Advances in neonatal intensive care have improved neonatal prognosis, but HIE survivors often face lifelong cerebral sequelae, such as developmental delay, cognitive and behavioral dysfunctions, cerebral palsy, epilepsy, and autism. There are few treatment options for HIE other than therapeutic hypothermia. Restoration of blood flow to ischemic lesions was proposed as an effective treatment but reperfusion can lead to additional injury through ischemic-reperfusion syndrome [1]. Experimental trials have demonstrated that pharmacologic agents and cerebral hypothermia may inhibit neonatal brain injury [2,3] and induce functional regeneration of damaged neural structures [4]. Although these are effects which reduce the morbidity of HIE, they are also limited. Mesenchymal stem cell (MSC) transplantation has recently been attempted to repair damaged brain tissues [5,6]. Unfortunately, MSCs take a long time to be recruited to ischemic lesions, reducing the potential for tissue repair. Furthermore, the ethical concerns of stem cell transplantation can pose challenges.

Stromal cell-derived factor-1α (SDF-1α) is constitutively expressed in endothelial, dendritic, stromal, and other cells. As a powerful chemokine, SDF-1α binds to Chemokine (C-X-C motif) receptor type 4 (CXCR4) and plays an important role in the regulation of cell motility [7]. SDF-1α can induce CXCR4^+^ immune cell accumulation at inflammatory lesions and CXCR4^+^ stem/progenitor cells during embryogenesis and tissue regeneration [8]. SDF-1α exerts pleiotropic effects on stem/progenitor cell trafficking. Moreover, SDF-1α at damaged tissues is reported to guide CXCR4^+^ MSCs to areas of the myocardial infarction [9].

Despite considerable work revealing the roles of SDF-1α in CXCR4 regulation, several aspects, such as temporal regulation, remain unknown. Although SDF-1α levels peak seven days after an injury, the increased presence of MSCs in damaged tissue is greatest from days 14 to 21 post injury. In addition, myocardial injection of SDF-1α into the area surrounding experimentally induced infarct areas significantly enhances myocardial endothelial progenitor cell density, vasculogenesis, and capillary density. However, it is unclear whether SDF-1α augments myocardial function by enhancing perfusion, reversing cellular ischemia, or increasing cardiomyocyte viability.

The above data suggest that SDF-1α may promote the repair of cerebral damage in neonatal HIE. However, the effects of SDF-1α in this process have not been fully elucidated. Therefore, the purpose of this study was to examine the effects of SDF-1α on brain damage in an HIE rat model.

## 2. Results

### 2.1. Magnetic Resonance Imaging (MRI)

**Figure 1:**
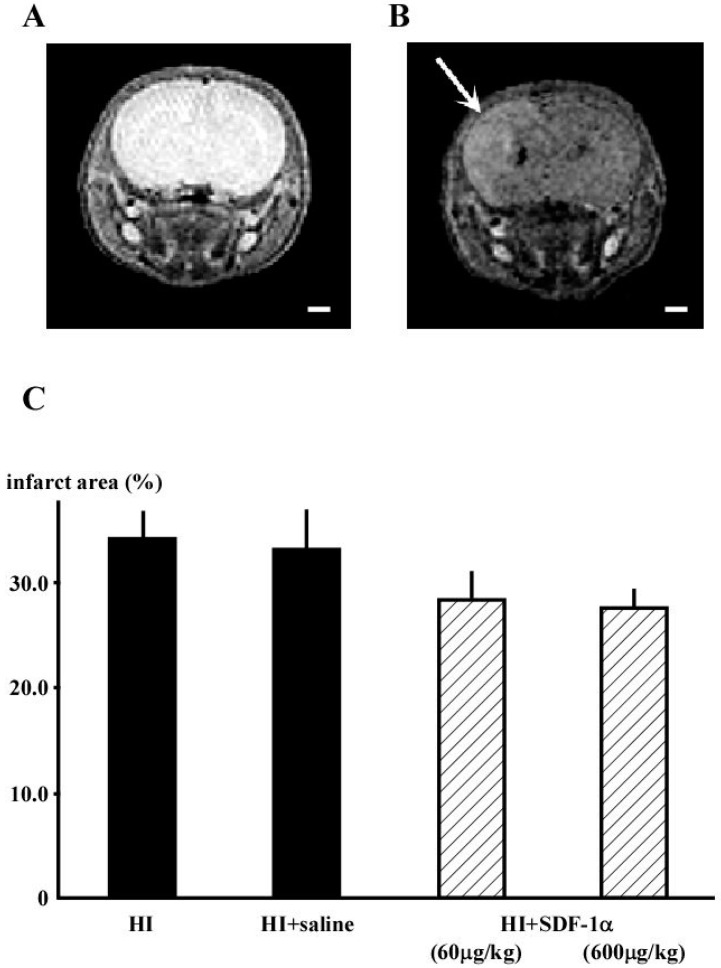
T2-weighted MRI images from P14 showing that surgery can produce HI in neonatal rat brains. Coronal cerebral sections from rats in the (**A**) sham and (**B**) HI groups. Infarction was observed in the WM (white arrow); and (**C**) Infarct area was slightly reduced by treatment with 600 μg/kg SDF-1α. Bar is 1mm.

Magnetic Resonance Imaging (MRI) was used to confirm that the experimental approach produced hypoxic ischemia (HI) in neonatal rat brains and to determine the therapeutic efficacy of SDF-1α treatment. T2-weighted MRI showed atrophy of the cortex cerebri and loss of white matter (WM). The ratios of the infarct area in the hemisphere ipsilateral to the carotid occlusion (%) were analyzed compared to those in normal rats (Figure 1A). The HI group demonstrated large infarct areas (33.8% ± 2.9%; *n* = 12; Figure 1B), which were slightly reduced by SDF-1α treatment (600 μg/kg; 27.8% ± 1.5%; *n* = 15). However, the infarct area after SDF-1α treatment was not different compared to that of the HI + saline group (33.3% ± 3.5%; *n* = 11; Figure 1C).

### 2.2. Morris Water Maze (MWM) Test

**Figure 2:**
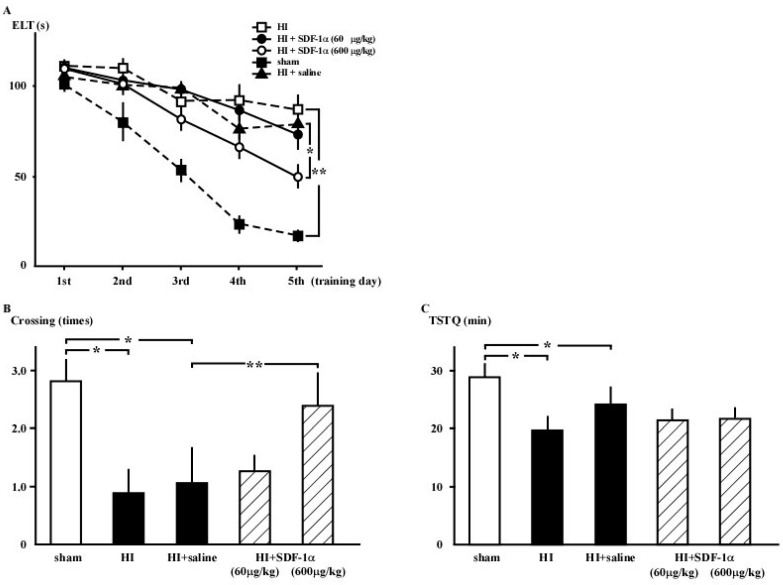
MWM test results, demonstrating that surgery disturbs spatial learning and memory in neonatal rats. (**A**) Sham group: filled squares with a dotted line. HI group: open squares with a dotted line. HI + SDF-1α (60) group: Solid circles with a solid line. HI + SDF-1α (600) group: open circles with a solid line. The ELT was significantly decreased by training and inhibited in the HI group compared to the sham group (** *p* < 0.01). ELT was restored by intracranial injection of 600 μg/kg SDF-1α (* *p* < 0.05); (**B**) Number of crossings was significantly decreased in the HI group (*n* = 14; * *p* < 0.005). Intracranial injection of 600 μg/kg SDF-1α (*n* = 15) mitigated these effects (** *p* < 0.05* vs.* HI + saline group); and (**C**) The TSTQ was significantly decreased in the HI group (*n* = 14; * *p* < 0.05), but impairment was not improved by SDF-1α.

The escape latency time (ELT) gradually decreased over the training period for all groups (Figure 2A). On day 5, the ELT in the HI group (87.9 ± 8.3 s, *n* = 8) was elevated compared to the sham group (17.6 ± 2.4 s; *n* = 8; *p* < 0.001). Treatment with SDF-1α (600 µg/kg) shortened the ELT (41.9 ± 10.7 s; *n* = 14; *p* < 0.05) compared to the HI + saline group (85.7 ± 14.7 s; *n* = 12). Fewer crossings were observed in the HI group (0.9 ± 0.4 times; *n* = 14) compared to the sham group (2.8 ± 0.4 times; *n* = 12; *p* < 0.005; Figure 2B). The number of crossings were increased by treatment with 600 µg/kg of SDF-1α (2.4 ± 0.6 times; *n* = 15) compared to the HI + saline group (1.1 ± 0.6 times; *n* = 12; *p* < 0.05). The mean time spent in the target quadrant (TSTQ) was also decreased in the HI group (19.8 ± 2.3 s; *n* = 14) compared to the sham group (28.8 ± 2.4 s; *n* = 12; *p* < 0.001), although SDF-1α treatment did not change the TSTQ (Figure 2C).

### 2.3. Rotarod Test

**Figure 3:**
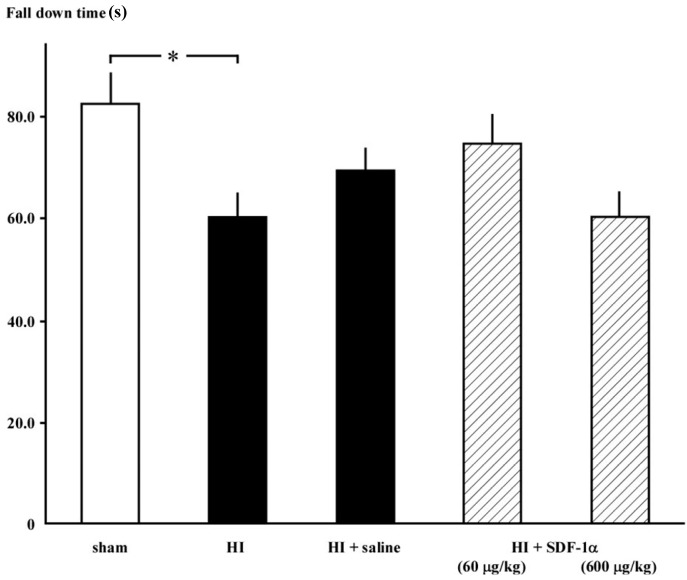
Rotarod test results demonstrating that motor coordination was disturbed in rats in the HI group. Fall-down time was decreased in the HI group compared to the sham group (* *p* < 0.05) and not affected by SDF-1α treatment.

The fall down time was decreased in the HI group (59.9 ± 5.0 s; *n* = 14; *p* < 0.05) compared to the sham group (81.9 ± 6.7 s; *n* = 13). 60 μg/kg SDF-1α slightly increased the fall down time; however, the difference was not statistically significant (Figure 3).

### 2.4. Staining with 2% 2,3,5-Triphenyltetrasodium Chloride (TTC)

**Figure 4:**
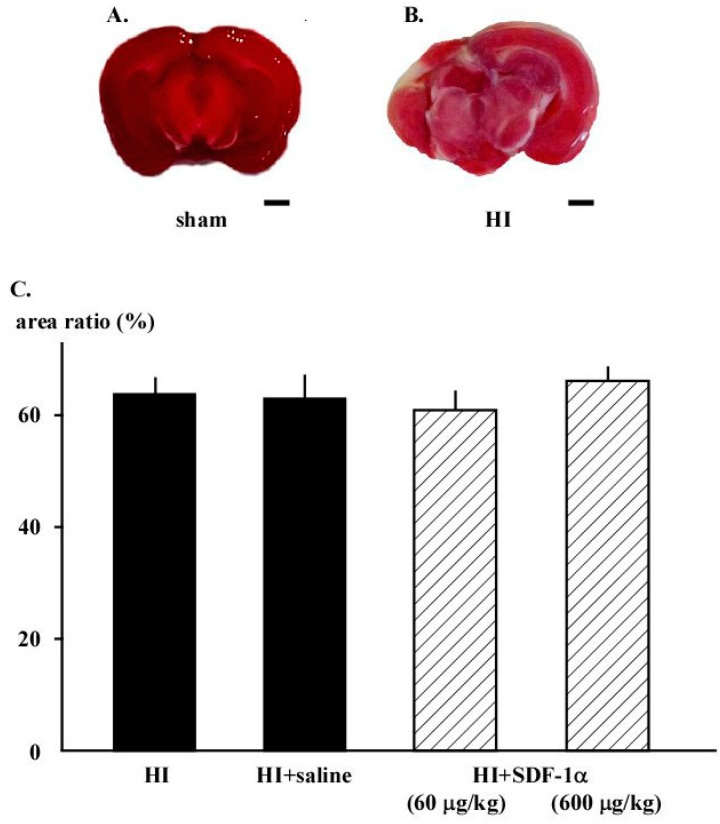
TTC staining reveals infarction and necrosis in the cerebral WM and around the hippocampus of animals in the HI group. (**A**) Neonatal rat brain in sham group demonstrates normal appearance; (**B**) Cerebral infarction was demonstrated in the ligated side of the brain as an atrophic and destructive area; and (**C**) SDF-1α did not change the infarct area in HI group. Bar is 1 mm.

TTC staining confirmed that the experimental protocol produced cerebral infarction in neonatal rats brains. Infarction and necrosis were observed in the cerebral WM and hippocampus in the HI group (Figure 4C). The total area of the infarct was high in the HI group (64.1% ± 2.9%) and was not affected by 60 or 600 μg/kg SDF-1α (66.2% ± 3.0%) (Figure 4).

### 2.5. Immunohistochemistry

**Figure 5:**
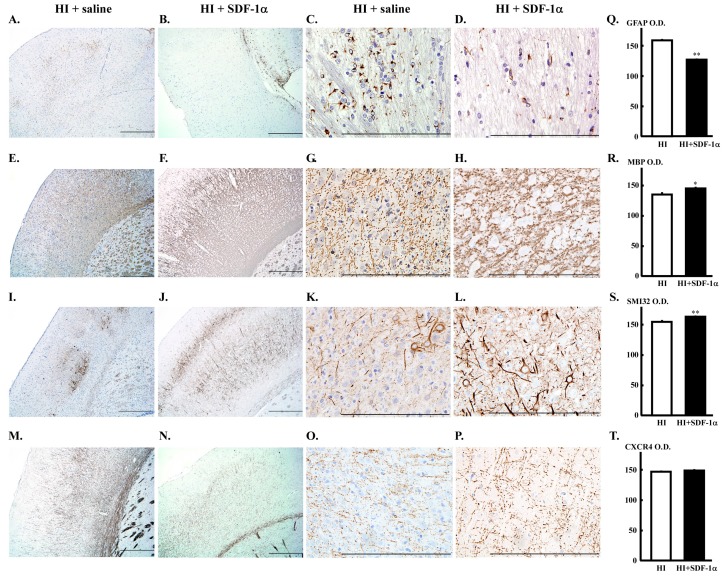
Immunohistochemistry of neonatal rat brains after HI. (**A**–**D**,**Q**) Representative cerebrum sections stained with anti-GFAP. Increased GFAP expression in WM was decreased by treatment with 600 µg/kg SDF-1α. Quantification data for each marker are presented. ** *p* < 0.01* vs.* HI group; (**E**–**H**,**R**) Representative cerebrum sections stained with anti-MBP. Weak MBP immunostaining in WM of the HI group was increased by treatment with 600 µg/kg SDF-1α. * *p* < 0.05* vs.* HI group; (**I**–**L**,**S**) Representative cerebrum sections stained with anti-SMI 32. Weak SMI 32 immunostaining in WM increased by treatment with 600 µg/kg SDF-1α. ** *p* < 0.01* vs.* HI group; (**M**–**P**,**T**) Representative cerebrum sections stained with anti-CXCR4. Extensive CXCR4 immunostaining in WM was not changed by treatment with 600 µg/kg SDF-1α. Bar is 500 µm.

The number of glial fibrillary acidic protein (GFAP)-positive cells was increased in the cerebral WM of the HI group (Figure 5A,C). Astrogliosis was significantly reduced by treatment with 600 μg/kg SDF-1α (Figure 5B,D,Q, ** *p* < 0.01* vs.* HI group). Myelin basic protein (MBP) showed weak immunostaining in WM in the HI group (Figure 5E,G). Treatment with 600 μg/kg SDF-1α significantly increased the size of the immune-stained area in the WM and increased myelination (Figure 5F,H,R, * *p* < 0.05* vs.* HI group). Staining for non-phosphorylated neurofilaments (SMI 32) was weak in the WM of the HI group (Figure 5I,K). Treatment with 600 μg/kg SDF-1α significantly increased the size of the immunostained area in WM-like filaments (Figure 5J,L,S, ** *p* < 0.01* vs.* HI group). CXCR4 immunostaining was observed throughout the WM of the HI group (Figure 5M,O), and was not changed by treatment with 600 μg/kg SDF-1α (Figure 5N,P,T).

## 3. Discussion

Transplantation of human umbilical cord blood mononuclear cells into neonatal rats with HIE stimulates neuronal differentiation, suppresses gliosis, and reduces behavioral deficits caused by brain injury [10]. Greggio* et al.* also reported that cell based-therapy using umbilical cord blood mononuclear cells prevented long-term cognitive deficits induced by neonatal HI [11]. However, in other studies, intra-venous [12] or intra-cardiac [13] infusion of the cells failed to lead to motor recovery in the rotarod test. Therefore, other methods to repair brain damage have been investigated. SDF-1α levels are increased in the ischemic brain of adult rats [14] indicating a potential therapeutic option. Data have shown that the injection of some chemokines can improve HI-induced neonatal brain injury [15]. Moreover, injection of SDF-1 into the penumbra was found to increase neovascularization around infarct areas [16,17]. In light of these findings, we investigated the therapeutic potential of intra-cranial injection of SDF-1α for neonatal HIE.

SDF-1α is a CXC chemokine that is produced by bone marrow stromal cells and a major chemoattractant for hematopoietic stem cells [18]. SDF-1α is expressed in many organs and binds mainly to CXCR4 [19,20,21]. CXCR4 is highly expressed in the central nervous system during embryonic brain development and also expressed in the adult brain. Because antibodies against CXCR4 can inhibit the migration of neural progenitor cells, the SDF-1α/CXCR4 system is thought to play important roles in the migration and proliferation of neural progenitor and stem cells to damaged areas of the brain [14,22,23]. Therefore, this system acts to regenerate cerebral tissue and to mitigate the effects of hypoxia-induced brain damage [24,25,26]. On the other hand, SDF-1 can also induce neuroprotection in hippocampal neurons [27] and stimulate the survival of Purkinje neurons [28]. Overall, the effects of SDF-1α can be divided into two types: (1) nervous system regeneration including repair by progenitor/stem cells and (2) neural functional protection. In the current study, SDF-1α could not repair the infarct lesion physiologically, although it provided some functional improvements. Therefore, the effect of SDF-1α in this study could be thought to be neural functional protection.

Manipulation of the SDF-1/CXCR4 axis could be beneficial in the early stages of nervous system injury, by combatting inflammation and apoptosis and by creating an environment for axon regeneration [29,30]. Balduini* et al*. reported that high neuronal plasticity could lead to the repair of motor coordination through restructuring of the HI-damaged neonatal brain [31]. Interestingly, our finding that motor coordination is not repaired by SDF-1α suggests that this molecule may not act as a neuromodulator in Purkinje cells in HI-injured neonatal brains. Instead, the data suggest that plasticity and synapse function may be repaired by SDF-1α, as memory and learning abilities were repaired by treatment.

The above results may coincide with the nature of SDF-1α effects. Indeed, the primary effects of HI (atrophy of the cortex cerebri and loss of WM) were not corrected, but secondary brain damage (neuronal degradation) [32] may have been affected. Published studies indicate SDF-1α may act by downregulating Bcl-2 and upregulating Bax [30], modulating inflammation [30,33], and increasing nitric oxide production by induced nitric oxide synthase [34]. Therefore, our data suggest that the neuronal protective functions of SDF-1α may focus on local areas of secondary brain damage.

In normal neural tissues, astrocytes regulate cerebral blood flow and synapse functions [35]. These cells are related to neuroprotection and reconstruction after brain injury [36]. However, reactive astrocytes can harm neural cells and astrogliosis can inhibit axon regeneration [37] by producing reactive oxygen, nitric oxide, and proinflammatory cytokines, such as tumor necrosis factor-α [38,39]. Ischemia induces astrogliosis around infarct areas [10]. Neuroinflammation can be evaluated by measuring astrocyte and microglial activation, as well as proinflammatory cytokine production, and may play important roles in the neuroinflammatory cascade in some diseases [40]. Periventricular leukomalacia (PVL) is associated with increased GFAP-positive astrocytes in the WM of neonatal brains [41]. Therefore, increased GFAP content in the neonatal rat brain is a potential indicator of the production of cytokines that can lead to PVL. Kim* et al.* reported that intracervical injection of lipopolysaccharide into pregnant rats induced hypomyelination, with suppressed expression of MBP, in the corpus callosum of a neonatal rat model of PVL [42]. The authors proposed that maternal inflammation-reduced MBP expression was caused by reduced MBP production by oligodendrocytes or a reduced number of oligodendrocytes. In this study, through GFAP staining, we demonstrated that SDF-1α could reduce HI-induced astrogliosis and lead to neural cells’ regeneration.

In addition to WM damage, HI-induced damage in the premature brain is characterized by deficient myelination [43,44]. Myelin deficiencies are involved in cognitive dysfunction [45] by affecting axonal function and neuronal survival [46]. These deficiencies can result in insufficient interconnections between cortical regions involved in information processing [47,48]. CA1 neurons in the hippocampus are also involved in cognitive functions such as spatial cognition [49].

In the current study, immunohistochemistry revealed that SDF-1α promoted remyelination through MBP, a protein located in the myelin membrane that leads to formation and stabilization of myelin. Immunohistochemistry of SMI 32 demonstrated that neurofilaments, neuronal cell bodies, dendrites, and axons were restructured by SDF-1α. These results indicate that SDF-1α could suppress the expression of proinflammatory cytokines in brain injury, thereby reducing secondary brain damage. Neuroinflammation can induce tissue destruction, resulting in loss of axons and myelin, and can cause chronic neurodegeneration with prolonged symptoms [50]. We propose that SDF-1α treatment improved cerebral function by reducing neuroinflammation without altering the cerebral infarct size.

Sun* et al.* reported a reduction in the endogenous expression of SDF-1α in damaged brain tissue [30]. Intracranial injection of recombinant SDF-1α reduced characteristics of traumatic brain damage including edema, serious dysfunction of the brain blood barrier, as well as apoptosis and degradation of neurons. These results support the idea that intracranial infusion of SDF-1α repairs secondary brain damage resulting in restored memory not but motor function.

SDF-1α is thought to induce neuroprotective effects in neonatal cerebral injury. Nevertheless, at the immature cerebral stage of the neonate, cell type- and region-specific effects could lead to marked dysregulation of crucial developmental processes, such as the migration and proliferation of neurons and glial cells. Given these limitations, further examination is needed to elucidate the mechanism of neonatal cerebral neuroprotection.

## 4. Experimental Section

### 4.1. Animals

All experiments were performed in accordance with institutional animal care guidelines and approval from the Ehime University School of Medicine. Wistar rat pups (Japan SLC, Inc., Hamamatsu, Japan) were housed under controlled conditions (constant temperature 24 ± 2 °C, 12-hour light/12-hour dark cycle) in cages with their dams until surgery. On postnatal day 7 (P7), surgery was performed on pups.

### 4.2. Neonatal HIE Animal Models

Hypoxic-ischemic brain injury was induced according to methods found in Rice *et al.* [51]. Pups were anesthetized with isoflurane (1.5%–3.5%; Pfizer, Inc., New York, NY, USA), and a small longitudinal cut was made along the midline of the ventral cervical skin. The left common carotid artery was ligated with 5–0 silk threads (Natsume Seisakusho Co., Ltd., Tokyo, Japan) and sectioned. At one to two hours after the surgery, animals were exposed to hypoxia (92% N_2_, 8% O_2_) for 2 h in a hypoxic chamber (Natsume Seisakusho) maintained at 36 °C (HI group). After hypoxia, pups were reanesthetized with isoflurane (1.5%–3.5%) and received intracranial injections of saline (HI + saline group; Wako Pure Chemical Industries, Ltd., Osaka, Japan) or SDF-1α in 0.1% bovine serum albumin (60 or 600 μg/kg, Sigma-Aldrich, St. Louis, MO, USA) (HI + SDF-1α (60), HI + SDF-1α (600) group). For the comparison group of HI group, sham operations were performed (sham). The surgery differing only in the absence of the ligation was conducted in the sham group. The left common carotid arteries were exposed but not ligated, and animals were not exposed to hypoxia.

Furthermore, mice were anesthetized with isoflurane, and the bregma was used for the puncture into the subdural space. A 30-gauge needle (TERUMO Corporation, Tokyo, Japan) was used to inject SDF-1α. This method was established by dye injections to ensure appropriate placement. The optimal dose of SDF-1α was determined by the data of the MWM test. A dose of 60 μg/kg SDF-1α decreased the ELT (73.3 ± 8.6 s) compared with the HI group (87.9 ± 8.3 s), but there was no statistical significance. A dose of 600 μg/kg SDF-1α (41.9 ± 10.7 s) significantly reduced the ELT. Therefore, we considered the optimal concentration of SDF-1α to be 600 μg/kg.

### 4.3. MRI

Seven days after HI (P14), all pups underwent MRI to identify the extent of brain injury. Pups were anesthetized with isoflurane (1%–3%) for the experiment. All scans were carried out on an MRI mini SA (DS Pharma Biomedical Co., Ltd., Osaka, Japan) and performed on the animal bed.

### 4.4. Behavioral Analyses

#### 4.4.1. MWM Test

Spatial perception was evaluated by the MWM test with a computerized video tracking system [52]. A black circular tank (150 cm diameter) was filled with water. A transparent platform was placed 1 cm below the water surface and maintained in the same location for all trials. Rats were trained five times per day at 15 min intervals for five consecutive days from P18 through P22 [53]. In each trial, rats were given 120 s to find the platform. Each trial consisted of a swim followed by a 10 s platform sit. On the fifth day, the hidden platform was removed, and the rats swam in the pool for 120 s. The number of times a rat crossed the former target position was measured. The ELT to locate the hidden platform in the water maze was taken as an index of acquisition or learning. The scene was video-tracked, and latency was recorded by using an EthoVision XT (Noldus Information Technology, Wageningen, The Netherlands). The mean time spent in all quadrants (TSAQ) and TSTQ were recorded. Rats that could not find the platform within 120 s were guided to it by the experimenter.

#### 4.4.2. Rotarod Test

Four weeks after HI (P35), the rotarod test was carried as described previously [54]. Briefly, rats were placed on a rotating horizontal plastic rod divided into four compartments (Rotarod model 47700, Ugo Basile, Italy). After three two-hour habituation sessions, every 2 h, each rat was given four trials with 15 min intervals. The rotarod was accelerated from four to 40 rpm for 2 min. The average time spent on the rod without falling down was recorded.

### 4.5. Assessment of Cerebral Injury

On P36, pups were sacrificed and used for TTC (Sigma-Aldrich) staining [55]. Brains were dissected and sliced into five coronal sections, with 2 mm thick, which were immediately incubated in 2% TTC in phosphate buffered saline (PBS) at room temperature (RT) for 20 min. TTC stained viable cells deep red as it was converted to red formazone pigment by nicotinamide adenine dinucleotide and lactate dehydrogenase. Infarcted cells had lost their enzymes and cofactors and, therefore, remained unstained. Cerebral infarct areas were compared between the ligated and the contra-lateral side. Infarct size was measured by Photoshop Extended (Adobe Systems Inc., San Jose, CA, USA).

### 4.6. Immunohistochemical Staining

On P36, immunohistochemical staining was performed. Rats were transcardially perfused with cold PBS (Sigma-Aldrich) followed by 4% paraformaldehyde (Electron Microscopy Science, Hatfield, PA, USA). Free-floating coronal brain sections were prepared at 40 μm of thickness in a sliding microtome (Brain matrices, EM Japan Co., Ltd., Tokyo, Japan). Immunolabeling was carried out by using a catalyzed signal amplification system (Dako’s Corp., Glostrup, Denmark), followed by blocking with 3% H2O2 for 5 min at RT and blocking buffer solution (30 min, RT). Blocked sections were incubated with primary rabbit antibodies for GFAP, MBP, SMI 32, CXCR4 (1:200, Bioss Inc., Woburn, MA, USA), or control IgG overnight at −4 °C. After washing in PBS, sections were incubated (1:1000) with appropriate biotinylated secondary antibodies for 30 min at 37 °C, followed by streptavidin-peroxidase complex for an additional 30 min. Labeling was visualized by reaction with diaminobenzidine. Sections were counterstained with hematoxylin. Staining intensity was quantified by acquiring 10 fields under high magnification on each slide with an Olympus microscope (BX50). Optical density (O.D.) was assessed by a public domain Java-based image-processing program (ImageJ version 1.49, National Institutes of Health, Bethesda, MD, USA).

### 4.7. Statistical Analyses

Data are reported as the means ± SE. Comparisons between groups were made by the Wilcoxon signed-rank test. TSTQ was analyzed by two-way ANOVA. Differences were considered to be significant at *p* < 0.05.

## 5. Conclusions

In brain injuries, the SDF-1α concentration around the infarct area is increased, leading to the promotion of functional recovery [56] and local cerebral blood flow [57]. SDF-1α can stimulate proliferation, survival, and maturation of oligodendrocyte progenitor and endothelial progenitor cells and promote remyelination and neovascularization through modulating the neuroinflammation [30,33]. In this study, SDF-1α repaired spatial perception and memory but not motor coordination through remyelination and axon regeneration. We conclude that SDF-1α represents a promising candidate for neonatal HIE therapy.
